# Enhancement mechanism of quantum yield in core/shell/shell quantum dots of ZnS–AgIn_5_S_8_/ZnIn_2_S_4_/ZnS†

**DOI:** 10.1039/d3na01052j

**Published:** 2023-12-28

**Authors:** Seonghyun Jeong, Minji Ko, Sangwon Nam, Jun Hwan Oh, Seung Min Park, Young Rag Do, Jae Kyu Song

**Affiliations:** a Department of Chemistry, Kyung Hee University Seoul 02447 Korea jaeksong@khu.ac.kr; b Department of Chemistry, Kookmin University Seoul 02707 Korea yrdo@kookmin.ac.kr

## Abstract

To achieve a high quantum yield (QY) of nanomaterials suitable for optical applications, we improved the optical properties of AgIn_5_S_8_ (AIS) quantum dots (QDs) by employing an alloyed-core/inner-shell/outer-shell (ZAIS/ZIS/ZnS) structure. We also investigated the mechanism of optical transitions to clarify the improvement of QYs. In AIS, the low-energy absorption near the band edge region is attributed to the weakly allowed band gap transition, which gains oscillator strength through state intermixing and electron–phonon coupling. The main photoluminescence is also ascribed to the weakly allowed band gap transition with characteristics of self-trapped excitonic emission. With alloying/shelling processes, the weakly allowed transition is enhanced by the evolution of the electronic structures in the alloyed core, which improves the band gap emission. In shelled structures, the nonradiative process is reduced by the reconstructed lattice and passivated surface, ultimately leading to a high QY of 85% in ZAIS/ZIS/ZnS. These findings provide new insights into the optical transitions of AIS because they challenge previous conclusions. In addition, our work elucidates the mechanism behind the enhancement of QY accomplished through alloying/shelling processes, providing strategies to optimize nontoxic QDs for various applications using a green chemistry approach.

## Abstract 2

The alloying/shelling processes enhance the weakly-allowed transition by the evolution of electronic structures and suppress the nonradiative process by the reconstructed lattice and passivated surface, ultimately leading to the high QY of 85%.

## Graphical abstract

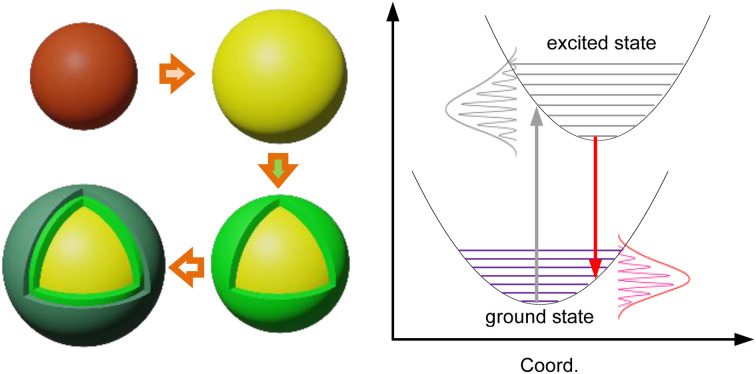

## 1. Introduction

Unique optical properties have been observed in quantum dots (QDs), making them promising candidates for applications in photocatalysis, solar cells, and light-emitting diodes.^1–3^ In addition to the widely investigated binary QDs, ternary QDs have gained attention due to their low toxicity.^4–10^ In addition, ternary QDs offer the facile tailoring of properties through adjustments of chemical composition, enabling control over the size and shape found in the binary QDs. Furthermore, significant improvements in photoluminescence (PL) quantum yield (QY) have been realized in ternary QDs over the past decade, showing the potential for bioimaging and light-emitting devices as alternatives to binary QDs.^8–10^ In particular, the introduction of alloyed core in core/shell structures of I–III–VI QDs has led to impressive QYs, with Cu–In–S QDs achieving a remarkable QY of 92% and Ag–In–S QDs exhibiting a QY of 87%.^11,12^ These QYs of I–III–VI QDs are comparable to the QYs of II–VI QDs.^13,14^ However, the underlying mechanism responsible for the enhanced QYs is still a subject of ongoing debate, because the optical processes have not been fully understood.

Ternary QDs of Cu–In–S and Ag–In–S, such as CuInS_2_ and AgInS_2_, commonly present broad PL bands with large Stokes shifts, which have been ascribed to defect-related emissions.^15–26^ The In-rich compound of Ag–In–S (AgIn_5_S_8_), denoted as AIS, also shows similar features. PL has been explained by the recombination of donor–acceptor pairs among various intragap states,^15–17^ while the recombination between the electrons in the conduction band and holes in acceptor-type states has also been proposed.^18–20^ Nevertheless, the broad band and large Stokes shift may not be attributable to defect states as similar characteristics of emissions suggest a specific type of defect states across QDs of different sizes and stoichiometries prepared using diverse methods. Hence, the concept of a self-trapped exciton has been introduced^27–29^ because it does not rely on a specific defect state to explain the broadband and large Stokes shift. In the self-trapped exciton model, the hole becomes localized on a small number of constituent elements even in defect-free lattice structures due to weak coupling between the constituents.^28,29^ Consequently, the localized hole induces lattice distortion, while the optical transitions are facilitated by electron–phonon coupling along the distorted coordinate, resulting in a broad band without the involvement of intragap states.^27,28^ However, this model could not elucidate the significant improvement of QYs observed in alloyed core structures.

The absorption of ternary QDs is typically weak and broad near the band edge region,^21–23^ which is recently described by the splitting of the valence band.^30,31^ According to the band splitting model, the optical transition from the odd-parity sublevel of the valence band to the lowest-lying conduction band is symmetry-forbidden, whereas the transition from the even-parity sublevel is allowed. In non-spherical QDs, however, the intermixing of the two sublevels induces the forbidden transition to gain intensity, leading to a weakly allowed transition from the odd-parity state.^22,28^ Therefore, absorption in the band edge region is feebly observed without a distinctive excitonic band, when the lowest-energy absorption is the weakly-allowed transition.^21–23^ The oscillator strength of the weakly-allowed transition depends on the dimensions of QDs, such as shape and size.^28,31^ Nevertheless, the effect of alloying on the electronic band structures has not been systematically investigated, although alloyed cores are expected to enhance the oscillator strength of the weakly allowed transition.

Previously, we incorporated Zn into AIS QDs to form the alloyed core (ZnS–AgIn_5_S_8_, ZAIS) and employed the multistep process to produce an inner-shell (ZnIn_2_S_4_, ZIS) and outer-shell (ZnS), to form ZAIS/ZIS/ZnS QDs with the QY of 85%.^12,32^ In these state-of-the-art QDs, two types of PL were attributed to the emissions of donor–acceptor pairs and surface-related defects. However, it remained unclear whether the defect states could entirely account for the substantial improvement in QY, even though the alloying/shelling process could enhance radiative processes and suppress nonradiative processes. In this study, we investigated the mechanism of optical transitions in AIS to clarify the improvement of QYs. The absorption band overlaps with the emission band, despite the large Stokes shift, which questions the interpretations of defect-related emissions. Accordingly, the low-energy absorption is proposed to be a weakly-allowed band gap transition, which gains oscillator strength through the state intermixing and electron–phonon coupling. The main emission was also characterized as the weakly allowed band gap transition with the features of self-trapped exciton. In alloyed cores, the weakly allowed transition was enhanced by the evolution of the electronic structures, which improved the band gap emission. In shelled structures, the nonradiative process is reduced by the reconstructed lattice and passivated surface, resulting in a high QY. Our results indicate the weak but clear band gap transitions, which differ from the previous conclusions,^15–26^ and thus propose new insights into the optical transitions of AIS. Hence, the improvement in QYs is mainly attributed to the enhanced band gap transitions achieved through the alloying/shelling processes.

## 2. Experimental section

### 2.1. Synthesis of QDs

Since the synthetic procedures have been previously described,^12,33^ a few key steps are briefly described here. To prepare AIS, silver nitrate and indium acetylacetonate were dissolved in a mixture of oleic acid (OA) and 1-octadecane (ODE) under N_2_ gas purging at room temperature. After 20 min, 1-octanethiol (OTT) was injected at 90 °C and reacted for 30 min. Subsequently, sulfur dissolved in oleylamine (OLA) was injected at 120 °C and reacted for 3 min to produce AIS QDs. For the first alloying/shelling process, zinc acetate dihydrate dissolved in OTT and OA was injected into the preformed QDs and the solution was maintained at 180 °C for 2 h. The second alloying/shelling process was also carried out by the injection of zinc acetate dehydrate, which was maintained at 230 °C for 2 h. An additional process was performed by injecting zinc acetate dehydrate, which was maintained at 180 °C for 2 h to form ZAIS/ZIS/ZnS QDs. The synthesized QDs were purified by centrifugation and subsequently stored in hexane for further characterization.

### 2.2. Characterization of QDs

The chemical compositions were examined using energy dispersive spectroscopy (EDS, JSM7401F). The crystal structures were determined by X-ray diffraction (XRD, D-max 2500) with Cu Kα radiation. The size and lattice were examined using transmission electron microscopy (TEM, JEM-2100F). UV-visible spectrometry (S-3100) was employed to measure the absorption spectra. The steady-state and time-resolved PL spectra were obtained using a homemade spectrometer.^34–36^ The QDs were excited by a second harmonic (360 nm) of a cavity-dumped oscillator (Mira/PulseSwitch, 720 nm, 150 fs). The collected emission was spectrally resolved using a monochromator, detected using a photomultiplier, and recorded using a time-correlated single photon counter (PicoHarp). QY was estimated by comparing it with rhodamine 6G.

## 3. Results and discussion

**Fig. 1:**
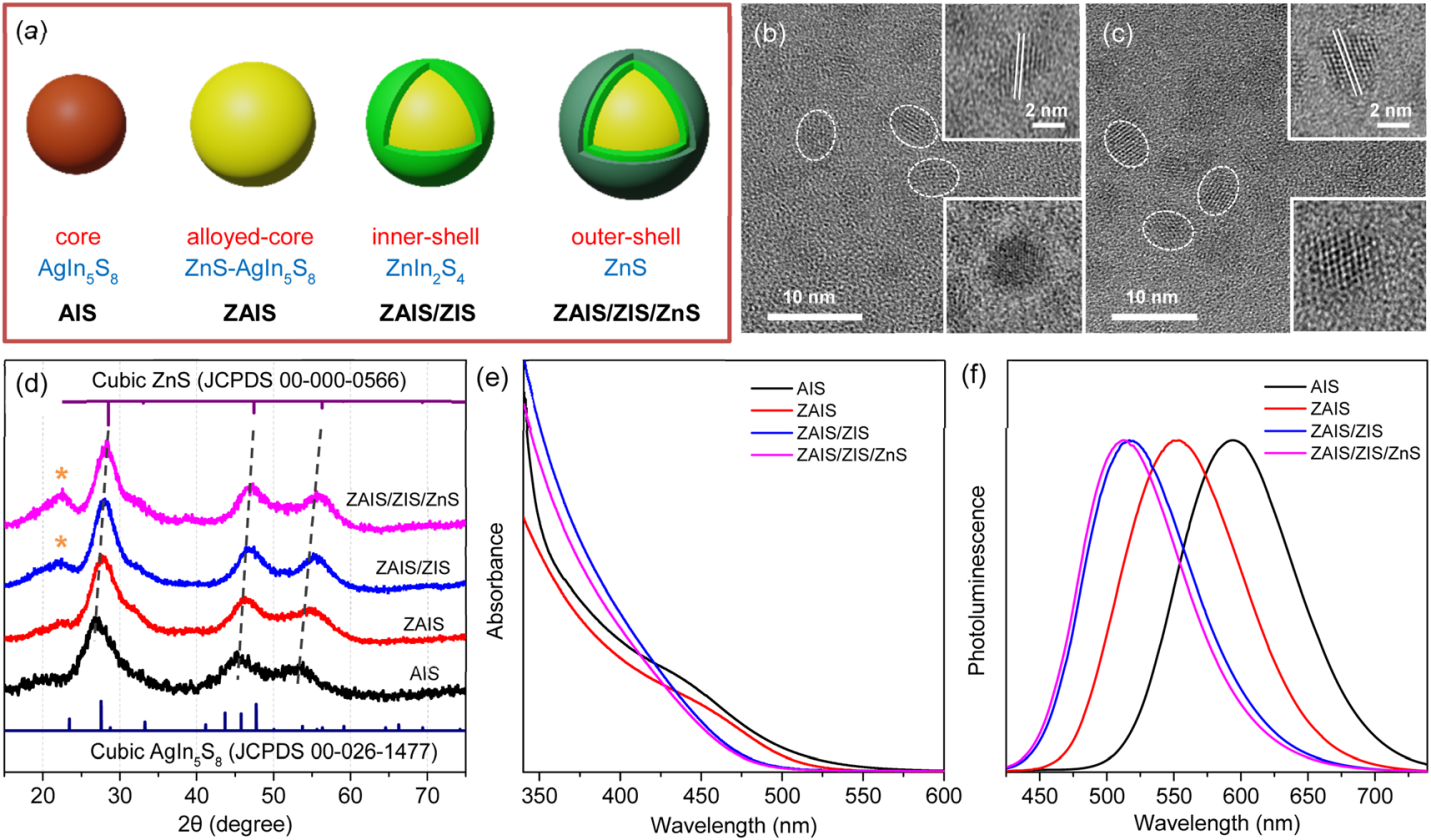
(a) Fabrication scheme to prepare ZAIS/ZIS/ZnS from AIS. TEM images of (b) AIS and (c) ZAIS/ZIS/ZnS. (d) XRD patterns of QDs. Asterisks indicate the (006) plane in the hexagonal phase of ZIS. (e) Absorption and (f) PL spectra of QDs.

QDs of the alloyed-core/inner-shell/outer-shell (ZAIS/ZIS/ZnS) were developed by the consecutive introduction of zinc acetate into the preformed QDs of AIS (Fig. 1a).^12,33^ These core/shell/shell structures exhibited a high QY (85%), comparable to that of the extensively studied Cd- and Pb-based QDs.^13,14^ TEM images revealed the spheroidal shapes of AIS (Fig. 1b) and ZAIS/ZIS/ZnS (Fig. 1c), indicating that well-faceted structures were nearly absent and size distributions were not trivial, as commonly observed in small anisotropic QDs.^4,28^ When averaged over 130 QDs (Fig. S1†), ZAIS/ZIS/ZnS (3.2 ± 0.4 nm) was slightly larger than AIS (2.8 ± 0.4 nm). The size of ZAIS/ZIS/ZnS includes the thickness of the inner-shell and outer-shell, in addition to the core, whereas the size of AIS is solely determined by the core. Therefore, the slight increase in size suggests the dynamic reactions of the QDs during the fabrication processes, such as etching, alloying, and shelling. The crystalline nature of ZAIS/ZIS/ZnS differs from that of AIS, as indicated by discrete interplanar distances of 0.30 nm and 0.32 nm, respectively. This difference in lattice distance supports the formation of a distinct crystal structure, *i.e.*, the alloyed core.

The crystal structures were further analyzed by XRD. Despite the broad diffraction peaks caused by the small sizes of the QDs, the XRD pattern revealed the cubic spinel phase peaks of AIS (Fig. 1d).^33,37^ With the alloying/shelling process, the diffraction peaks of the AIS shift towards higher angles, approaching the cubic phase peaks of ZnS.^12,33^ This shift indicates the formation of a solid solution between AIS and ZnS. The diffraction peak at 22° corresponds to the (006) plane in the hexagonal phase of ZIS,^38,39^ suggesting the presence of a ZIS layer. Elemental analysis indicates an increase in the Zn ratio during the fabrication processes (Fig. S1†). However, it is difficult to precisely determine the Zn ratio in the core, because EDS results cannot distinguish Zn in the core (ZAIS) from Zn in the shell (ZIS and ZnS).^5,37^ Indeed, the elemental ratio in AIS and ZAIS/ZIS/ZnS deviates from the bulk stoichiometry, as frequently observed in the QDs of AIS.^37,40,41^ Overall, TEM, XRD, and EDS provide evidence of the core transition from AIS to ZAIS and the formation of shell layers during the fabrication processes.

The core transition and shell formation influence the optical features of the QDs, such as the blue-shift of the absorption tail (Fig. 1e). In fact, the observed shift of the onset in the long-wavelength region could be associated with the disappearance of Urbach absorption,^27,40^ due to annealing effects at high temperatures during fabrication processes. However, PL spectra exhibit an analogous shift (Fig. 1f) and the PL excitation spectrum shows a comparable feature to the absorption spectrum near the onset (Fig. S2†), indicating that the absorbing species mainly contribute to the emission process. Hence, the removal of intragap states (Urbach absorption) is not mainly responsible for the observed shift of the absorption tail. Hence, this shift indicates the evolution of electronic band structures through the development of the alloyed core.^41,42^ Nevertheless, the change of the band gap energy (*E*_g_) is not well correlated to the degree of alloying observed in the XRD patterns, when the *E*_g_ is estimated using the Tauc plot (Fig. S2†).^42,43^ Besides, the *E*_g_ of AIS (∼3.5 eV) appears too high compared to the typical quantum confinement effects, because the *E*_g_ of AIS with a diameter of 2.8 nm is expected to be in the range of 2.35–2.55 eV, when the electronic structures are calculated using a finite-depth-well effective-mass approximation.^32,44^ Therefore, Tauc analysis is not appropriate for an accurate estimation of *E*_g_ in the system of AIS, primarily because of the absence of a characteristic absorption feature.

**Fig. 2:**
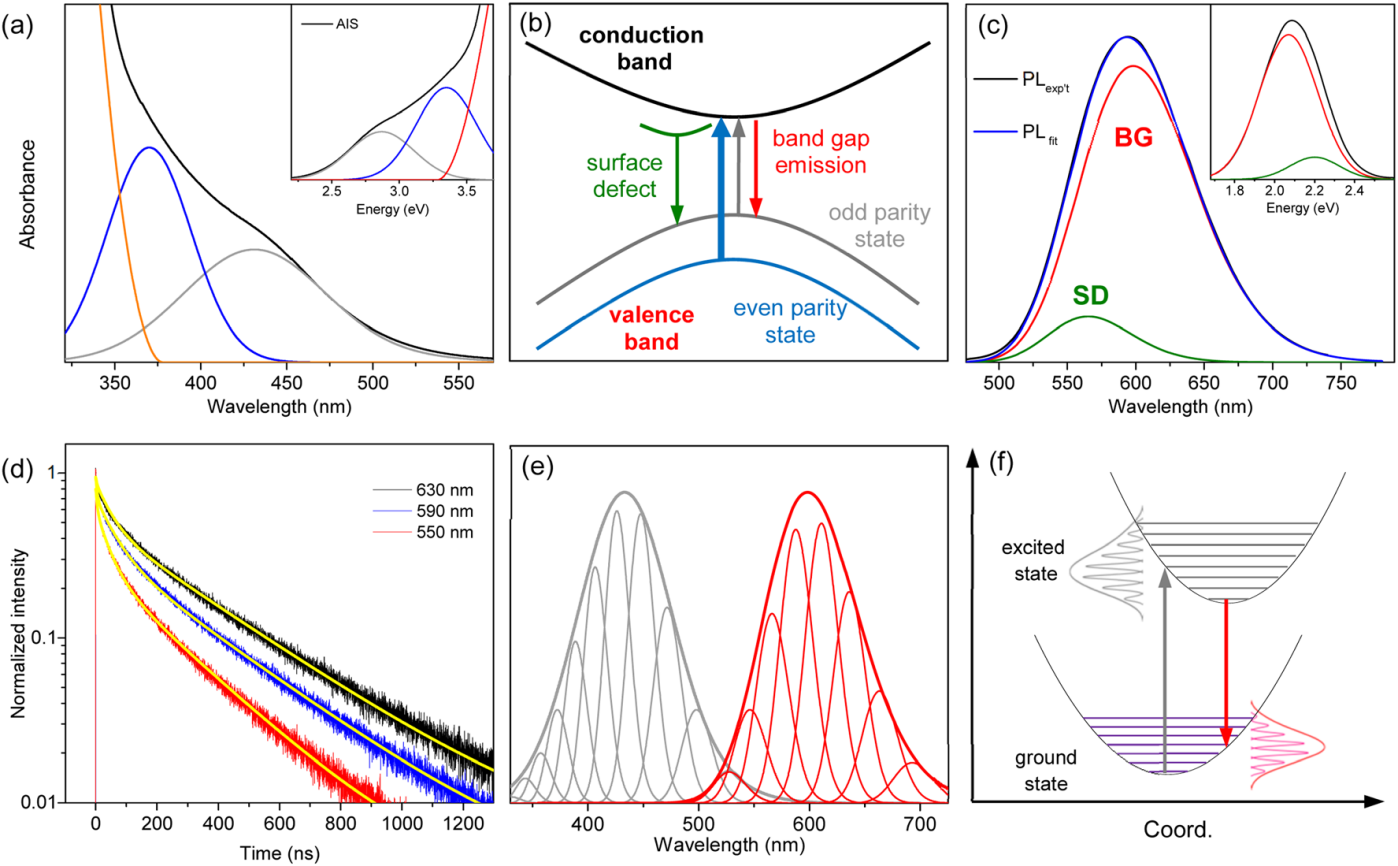
(a) The absorption spectrum of AIS is deconvoulted into two Gaussian bands and the increase of absorbance. The inset shows the deconvoulted spectrum in the energy scale. (b) Schematic band structure and optical transitions from the odd-parity and even-parity sublevels. (c) The PL spectrum of AIS is deconvoulted into two Gaussian bands (BG and SD). The inset shows the deconvoulted spectrum in the energy scale. (d) The time profiles of PL at different emission wavelengths are fitted by the bi-exponential model (yellow lines). (e) The low-energy absorption (gray) and BG (red) are deconvoulted into phonon modes, which are broadened to reproduce the spectral profiles. (f) Schematic optical transitions along the coordinate coupled to the electronic transition. The absorption (gray arrow) and PL (red arrow) occur between the same pair of electronic states.

The hump in the absorption tail suggests an excitonic absorption despite its low intensity. Accordingly, the absorption of AIS is deconvoulted into Gaussian bands and a gradual increase in absorbance.^34,35^ The absorption spectrum obtained in the wavelength scale is converted to the energy scale to employ Gaussian functions (inset of Fig. 2a), which is converted again to the wavelength scale (Fig. 2a). Recent studies have proposed two types of optical transitions near the band edge region of I–III–VI QDs, arising from the splitting of the valence band into odd-parity and even-parity sublevels (Fig. 2b).^30,31^ In the band structure model,^31^ the low-energy transition involves the odd-parity sublevel with p-type symmetry (gray line) and the conduction band with s-type symmetry (black line), which is not formally allowed by the symmetry selection rule. However, in non-spherical QDs, this transition can be weakly allowed due to the intermixing of the two sublevels. Therefore, the low-energy (long-wavelength) absorption in the deconvoulted spectrum is ascribed to the weakly-allowed transition from the odd-parity state (gray arrow), which gains oscillator strength through state intermixing, as in CuInS_2_ and AgInS_2_.^28,45^ The other absorption at higher energy (short-wavelength) corresponds to the symmetry-allowed transition (blue arrow) from the even-parity sublevel with s-type symmetry (blue line) to the conduction band with s-type symmetry (black line), showing higher absorbance due its higher oscillator strength. Although the band structures of AIS have not been reported, the low-energy absorption of AIS shows the characteristics of the weakly allowed transition, suggesting similar band structures to CuInS_2_ and AgInS_2_.

The PL spectrum of AIS is slightly asymmetric (Fig. 2c), which can be deconvoluted into two Gaussian bands in the energy scale (inset of Fig. 2c). These bands are assigned as the emissions of the surface-related defects (SD) and band gap states (BG), as discussed in the following sections. The time-resolved PL (TRPL) supports the presence of two emissive states because the time profiles are well fitted by a bi-exponential model with lifetimes of *τ*_1_ = 48 ± 5 ns and *τ*_2_ = 320 ± 10 ns (Fig. 2d). The relative magnitudes of these two components vary with the emission wavelength, indicating a wavelength-dependent contribution of the two states. The fast component is associated with the short-wavelength band (SD) because its relative magnitude decreases with increasing wavelength. The slow component with the major contribution in the entire wavelength region is attributed to the main emission (BG). In TRPL spectra, which are reconstructed from the time profiles at intervals of 5 nm (Fig. S3†), the band shape changed with the detection time, supporting a time-dependent contribution of the two states. Certainly, the integrated TRPL spectrum for the temporal range of 0–2000 ns is nearly identical to the steady-state PL spectrum, suggesting that the relaxation processes are mostly completed in 2000 ns. Notably, the band shape of BG agrees well with the TRPL spectrum at 2000 ns, where the contribution of SD is expected to be minimal by a much shorter lifetime than BG, providing another evidence for the decomposition process into BG and SD.

BG shows a large Stokes shift (Fig. S4†), reaching up to 230 nm (∼1.3 eV) compared to the high-energy absorption (symmetry-allowed transition). The Stokes shift decreases to 165 nm (∼0.8 eV) when compared to the low-energy absorption (weakly-allowed transition), which is still larger than the typical Stokes shift observed in QDs (<0.1 eV).^13,14^ Besides, the bandwidth of BG (∼100 nm) is much broader than the ordinary width of band gap emission in QDs (<30 nm).^1,13^ To elucidate the origin of the large Stokes shift and broad emission band, several possibilities have been proposed, although the underlying mechanism remains a subject of discussion.^15–26^ It is often attributed to the Coulomb interactions of the donor–acceptor pairs, while the coexistence of multiple defect states is also suggested. In addition, the self-trapped exciton, where the hole is localized on small moieties, has been proposed in the QDs of CuInS_2_ and AgInS_2_.^27–29^ The localization of the hole would induce the lattice distortion, which leads to electron–phonon coupling along the coordinate of distortion. The emission energy (*E*_em_) is thus correlated to the degree of phonon coupling.

1 *E*_em_ = *E*_ZPL_ − *nħω*

Here, *E*_ZPL_ represents the energy of the zero-phonon line, *n* is the number of phonons, and *ħω* is the phonon energy. Since the A_1_ mode (305 cm^−1^) and E_LO_ mode (362 cm^−1^) are predominantly active among the optical phonon modes of AIS,^46^ a combination of these two modes is tentatively employed to evaluate the contribution of phonon coupling.^34,35^ The spectrum in the energy scale is deconvoulted into symmetric Gaussian functions (Fig. S4†), which is also presented in the wavelength scale (Fig. 2e). The analysis shows the *E*_ZPL_ value of 2.35 eV in BG, when the energy and FHWM of the phonon mode are 0.08 and 0.12 eV, respectively.

The strength of the electron–phonon coupling is then estimated using the configuration coordinate model,^27,34^2
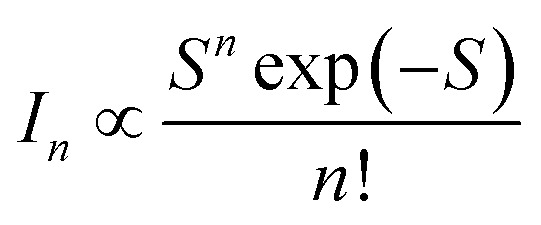
The intensity of the phonon lines (*I*_*n*_) is described by the Huang–Rhys factor (*S*), which represents the average number of phonons involved in the transition. The deconvoulted bands are simulated with *S* = 4.1 (Fig. S4†), suggesting a substantial contribution of phonon modes presumably due to an extensive change in geometry between the ground and excited states.^47,48^ The slight deviation of the simulation from the experimental result could be attributed to the size distribution of the QDs, as well as the assumption of the combination mode. Nevertheless, the emission band could not be well described by a single phonon mode (Fig. S4†), indicating that electron–phonon interactions occur in multiple phonon modes.^47,48^ Hence, the broad emission band can be ascribed to the presence of phonon replicas, although a comprehensive analysis of phonon modes requires further investigation.

Since the absorption band shows comparable broadness to the emission band (Fig. 2e), the absorption band is also analyzed using the phonon-dependent energy (*E*_ab_).

3 *E*_ab_ = *E*_ZPL_ + *nħω*

The low-energy absorption is deconvoulted with *E*_ZPL_ of 2.35 eV and *ħω* of 0.13 eV (Fig. S4†). The *E*_ZPL_ represents the lowest-energy mode in the absorption and thus the *E*_g_ of the QDs, which is in agreement with the expected values obtained from the effective-mass approximation (2.35–2.55 eV). Although the *E*_ZPL_ is identical in the deconvoulted absorption and emission spectra, the phonon energies are distinct, which can be correlated to a difference in the shape of the potential energy surface between the ground and excited states (Fig. 2f).^47,48^ The Huang–Rhys factor in the absorption process (*S* = 4.1) is equivalent to that in the emission process, indicating that both absorption and emission are associated with the same pair of states (Fig. 2f) because the Huang–Rhys factor is normally determined by the difference in the equilibrium geometry between the two states. Besides, the long-wavelength tail of the absorption band (low-energy transition) overlaps with the short-wavelength tail of the emission band (BG), despite the large Stokes shift (Fig. 2e), confirming that both optical transitions occur between the same pair of states with the identical *E*_ZPL_.

The low-energy absorption is supposed to acquire oscillator strength from the allowed transition, although the borrowed intensity is influenced by the size and shape of the QDs.^28,31^ Furthermore, the phonon interactions coupled with the optical transition enhance the oscillator strength.^47,48^ Accordingly, the low-energy absorption represents the lowest-energy band gap transition, despite the weak intensity and large bandwidth. Likewise, BG corresponds to the band gap transition with the identical *E*_ZPL_ and Huang–Rhys factor to the low-energy absorption. Although efficient absorption may occur to a higher-lying excited state, the emission mainly occurs from the lowest-excited state, due to the fast relaxation process (Fig. S5†), implying that BG is the band edge transition. Previous studies have mostly attributed the emission of AIS to defect states,^15–26^ due to the large Stokes shift and broad band. However, the self-trapped exciton can explain the large Stokes shift and broad band through phonon coupling even in defect-free structures.^27–29^ In the self-trapped exciton, a few silver elements could introduce localized orbitals just above the typical valence band, due to weak coupling between them, which governs the optical features. The increase in the silver element increases the number of silver-based orbitals but does not substantially alter their energies. Consequently, the optically active orbitals remain almost unchanged with the moderate change of the silver ratio, accounting for the equivalent optical properties of AIS, despite the deviation from bulk stoichiometry (Fig. S1†). Besides, the weakly-allowed nature of BG, which is the reverse transition of the low-energy absorption, accounts for its long lifetime (320 ns). Therefore, the common features of the optical transitions, such as the large Stokes shift, broad band, and long lifetime, are correlated to the weakly-allowed, phonon-assisted transition of band gap states, supporting the band edge emission of BG.

The other emission band with a lower intensity (SD) is supposed as the emission of surface-related defects because the energy and lifetime of SD are comparable to those of the surface states.^43,49^ The emission energy of SD seems to be higher than that of BG at first glance (Fig. 2c). However, when the emission of SD is deconvoulted into a few phonon modes with the same energy and width as the emission of BG (Fig. S5†), the *E*_ZPL_ of SD (2.30 eV) is lower than that of BG (2.35 eV). Since the similar energy and width of phonon lines allow the comparison of the emission bands, the *E*_ZPL_ indicates that SD is correlated to the shallow defect states on the surfaces. In other words, the energy difference (0.05 eV) implies a partial loss of energy upon trapping at surface defect states.^43,49^

**Fig. 3:**
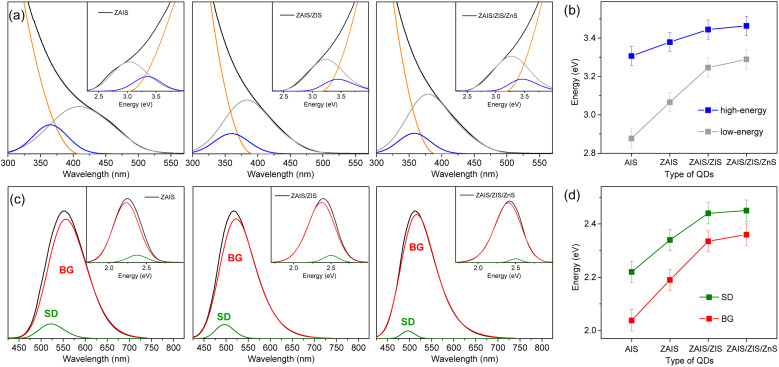
(a) Absorption spectra of QDs are deconvoulted into two Gaussian bands and the increase of absorbance. The insets show the deconvoulted spectra in the energy scale. (b) Peak energies of high-energy and low-energy absorption with fabrication processes. (c) The PL spectra of QDs are deconvoulted into two Gaussian bands (BG and SD). The insets show the deconvoulted spectra in the energy scale. (d) Peak energies of BG and SD.

The fabrication process induces a blue-shift in the optical transitions, indicating an increase in the *E*_g_ of QDs. When the absorption profiles are deconvoulted from the monotonous increase in absorbance (Fig. 3a), the transition energies of the two absorption bands exhibit a characteristic blue-shift (Fig. 3b), in contrast to the estimated energies using the Tauc plot (Fig. S2†), supporting the influence of alloying.^41,42^ Remarkably, the low-energy absorption becomes more pronounced with fabrication processes, inferring the enhancement of the state intermixing through the evolution of electronic structures with alloying.^28,31^ The fluctuation of the composition in the alloyed core may further relax the symmetry selection rule, thus improving the oscillator strength. Likewise, BG is enhanced by alloying (Fig. 3c). The blue-shift of BG is consistent with that of the low-energy absorption (Fig. 3d), providing additional evidence for the correlated transitions between the low-energy absorption and BG. On the other hand, the intensity of SD decreases with the fabrication processes, possibly due to the reconstructed lattice by annealing and the passivated surface by the shell layers.^43,49^ The time profiles support a reduced contribution of SD because the magnitude of the fast component decreases (Fig. S6†). The blue-shift of SD is different from that of the low-energy absorption and BG (Fig. 3d) because defect states are less correlated to the evolution of the electronic structures. The change of intensity and energy with the fabrication processes thus confirmed that SD is ascribable to the defect states.

**Fig. 4:**
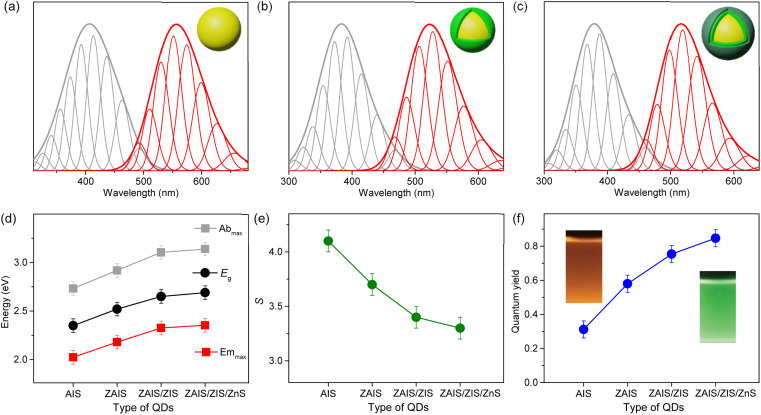
Low-energy absorption (gray) and BG (red) in (a) ZAIS, (b) ZAIS/ZIS, and (c) ZAIS/ZIS/ZnS are deconvoulted into progression of phonon modes. (d) Energies of absorption maximum (Ab_max_), emission maximum (Em_max_), and band gap energy (*E*_g_) of QDs. (e) Huang–Rhys factor (*S*) of optical transitions in QDs. (f) Quantum yield of QDs.

The shapes of the low-energy absorption are similar to those of BG in the alloyed cores, while both optical bands are still broad (Fig. 4). When the optical bands are deconvoulted into the phonon modes (Fig. S7†), the absorption and emission of the alloyed cores show the identical *E*_ZPL_ (Fig. 4d), indicating the band gap transitions between the same pair of states. The Huang–Rhys factor becomes reduced with fabrication processes (Fig. 4e), although it remains nontrivial in ZAIS/ZIS/ZnS (Fig. S8†), leading to the decrease in the Stokes shift (Fig. 4c). Accordingly, we propose that the band gap transition of the alloyed core also gains intensity through the state intermixing and phonon coupling, while the decrease in the Huang–Rhys factor implies a reduced contribution of phonon coupling. Therefore, the enhanced oscillator strength in the alloyed core can be primarily ascribed to the evolution of the electronic structures,^21,41^ which contributes to the improvement of the QY in ZAIS/ZIS/ZnS (Fig. 4f).

To investigate the improvement of QY in more detail, the relaxation processes are examined, because QY is determined by the radiative and nonradiative relaxation.4
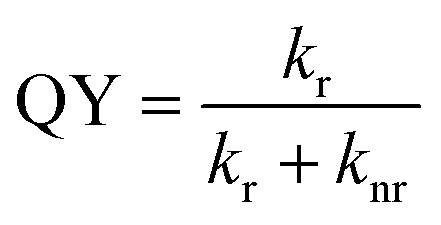
Here, *k*_r_ and *k*_nr_ are the radiative and nonradiative rates, respectively, which also determine the lifetime (*τ*) of an excited state.5
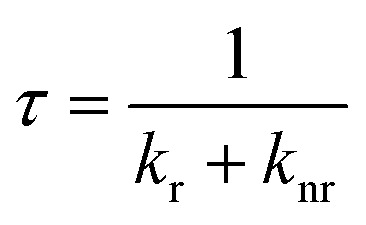
Since it is difficult to precisely separate the QY of band gap emission from that of the surface emission, we assume the measured value as the QY of band gap emission. Indeed, the intensity of SD is low (<10%), which would minimally influence the estimation process. In the BG of AIS with *τ* of 320 ns and QY of 31%, *k*_r_ and *k*_nr_ are calculated to be 0.97 × 10^6^ s^−1^ and 2.16 × 10^6^ s^−1^, respectively.^32,35^ Similarly, in the BG of ZAIS/ZIS/ZnS with *τ* of 410 ns and QY of 85%, *k*_r_ and *k*_nr_ are 2.07 × 10^6^ s^−1^ and 0.37 × 10^6^ s^−1^, respectively. Compared to AIS, *k*_r_ increases substantially in ZAIS/ZIS/ZnS (210%). The alloyed core improves the radiative process because the electronic structures of ZAIS enhance the oscillator strength of the band gap transitions.^21,41^ Besides, *k*_nr_ is significantly suppressed (20%), due to the reduced defects by the reconstructed lattice and passivated surface.^43,49^ The inner shell of ZIS, acting as the interface layer between ZAIS and ZnS, also plays a role in suppressing the nonradiative process.^12,32^ The ZIS layer could alleviate lattice strain arising from the unmatched crystal structures between ZAIS and ZnS, facilitating the gradual release of strain to reduce the nonradiative process.^50,51^ Overall, through the alloying/shelling processes, radiative processes are enhanced by the evolution of the electronic structures and nonradiative processes are reduced by the reconstructed lattice and surface, ultimately leading to high QY of ZAIS/ZIS/ZnS.

It is noted that the two types of absorption in AIS have been previously attributed to the band gap and intragap transitions.^27,40^ However, in the current study, we suggest that both absorptions are the band gap transitions, which originate from the two sublevels of the valence band, as in CuInS_2_ and AgInS_2_.^28,45^ Specifically, the low-energy absorption is ascribed to the weakly-allowed band gap transition from the odd-parity state of the valence band, rather than the intragap transition, although the calculational and experimental evidence are further required to confirm the contribution of the odd-parity state. Moreover, we question the conventional understanding of the broad emission in AIS, which has been correlated to the defect states.^15–26^ We propose that the major emission is the band gap transition with the nature of self-trapped exciton, as often suggested in CuInS_2_ and AgInS_2_.^27–29^ The band gap emission to the odd-parity state is the reverse transition of the low-energy absorption with the properties of the weakly-allowed transition. In addition to the similar Huang–Rhys factors between the absorption and emission profiles, the long-wavelength tail of the absorption band overlaps with the short-wavelength tail of the emission band, confirming that both transitions occur between the same pair of states. Certainly, the optical features of AIS, such as the absorption and emission bands, are comparable to those of CuInS_2_ (Fig. S9†), where the low-energy absorption and main emission were attributed to the band gap transitions.^34,35^ Hence, the optical characteristics support a new model of the weakly allowed, phonon-assisted transition of the band gap states in AIS. These results further elucidate the improvement of QYs by the evolution of electronic structures attained through the alloying process, as well as by the reduced nonradiative processes in the reconstructed lattice and surface accomplished through the shelling process.

## 4. Conclusions

We improved the optical properties of the AIS QDs by employing the alloyed-core/inner-shell/outer-shell structure to achieve the QY of 85%. In this system of interest, we investigate the mechanism of optical transitions to clarify the enhancement of QYs. The low-energy absorption is ascribed to the weakly allowed band gap transition, which gains oscillator strength through the state intermixing and electron–phonon coupling. The main emission is also the weakly-allowed band gap transition, where the large Stokes shift and the broad band are explained by the self-trapped exciton model with phonon coupling. During the multistep fabrication, the core structures are alloyed and the shell structures are formed. In the alloyed core, the weakly allowed transition becomes enhanced by the evolution of electronic structures, which also improves the band edge emission. In the shelled structure, the nonradiative process is reduced by the reconstructed lattice and passivated surface. Therefore, the improved radiative process and suppressed nonradiative process lead to the high QY in ZAIS/ZIS/ZnS. Our findings challenge previous conclusions and provide an alternative scheme of the optical transitions in AIS, which elucidates the improvement of QYs and offers insights into the fabrication of QDs with a green chemistry approach.

## Conflicts of interest

There are no conflicts to declare.

## Supplementary Material

NA-006-D3NA01052J-s001
