# Supplementary material for: Enhancement mechanism of quantum yield in core/shell/shell quantum dots of ZnS–AgIn5S8/ZnIn2S4/ZnS
Source: Nanoscale Adv. 2023 Dec 28;6(3):925–33. doi: 10.1039/d3na01052j (PMC10825935; doi:10.1039/d3na01052j)
Supplement: NA-006-D3NA01052J-s001 [file NA-006-D3NA01052J-s001.pdf]

Electronic Supplementary Material (ESI) for Nanoscale Advances.
This journal is © The Royal Society of Chemistry 2023

Electronic Supplementary Information

Enhancement Mechanism of Quantum Yield in Core/Shell/Shell Quantum Dots of ZnS−$AgIn_5S_8$/$ZnIn_2S_4$/ZnS

Seonghyun Jeong,[‡a] Minji Ko,[‡b] Sangwon Nam,[a] Jun Hwan Oh,[b] Seung Min Park,[a] Young Rag Do*[b] and Jae Kyu Song*[a]

[a] Department of Chemistry, Kyung Hee University, Seoul 02447, Korea

[b] Department of Chemistry, Kookmin University, Seoul 02707, Korea

* Corresponding authors. E-mail addresses: yrdo@kookmin.ac.kr; jaeksong@khu.ac.kr

‡ S. J. and M. K. equally contribute to this work.

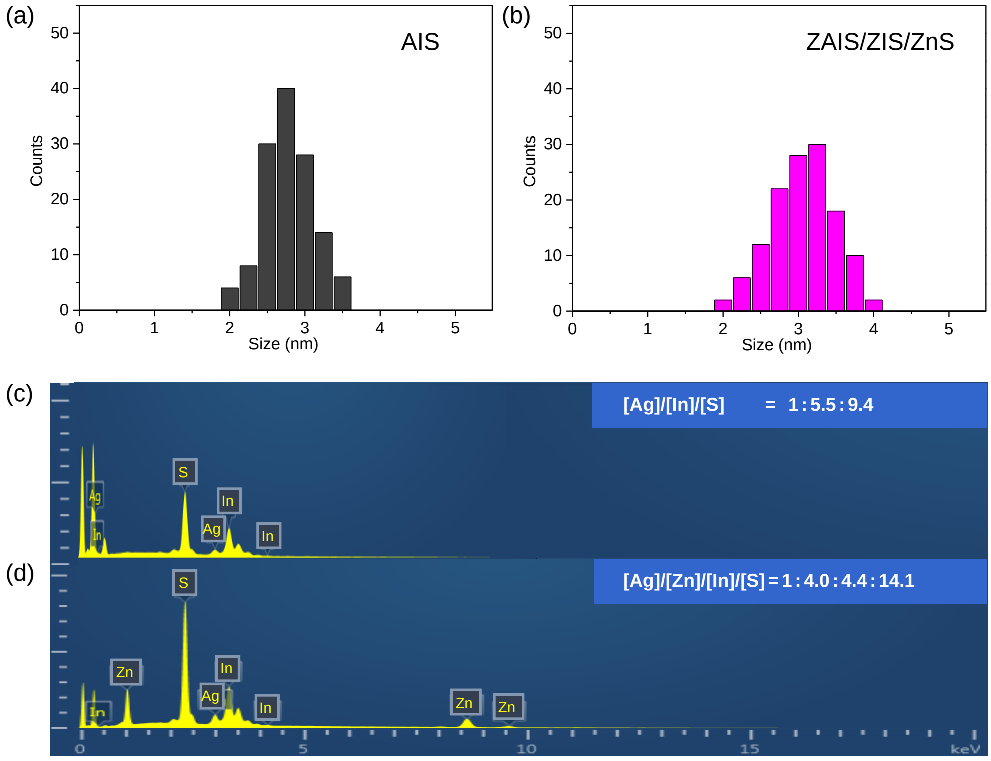

Fig. S1 Size distributions of (a) AIS and (b) ZAIS/ZIS/ZnS QDs measured by HRTEM. Chemical compositions of (c) AIS and (d) ZAIS/ZIS/ZnS obtained by EDS.

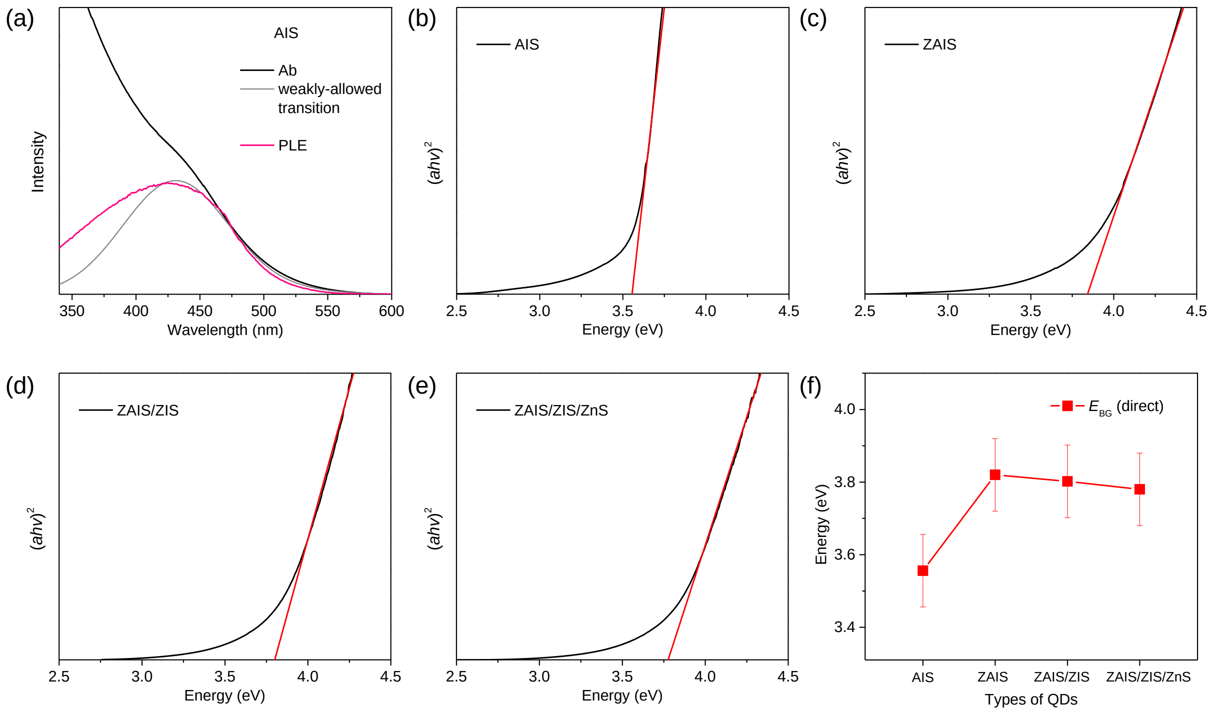

Fig. S2 (a) The PL excitation spectrum (pink) of AIS obtained at the maximum emission wavelength is compared to the absorption spectrum (black). The PL excitation shows the comparable feature to the low-energy absorption. The band gap energies of (b) AIS, (c) ZAIS, (d) ZAIS/ZIS, and (e) ZAIS/ZIS/ZnS are estimated using the Tauc plot. (f) Estimated band gap energies of QDs.

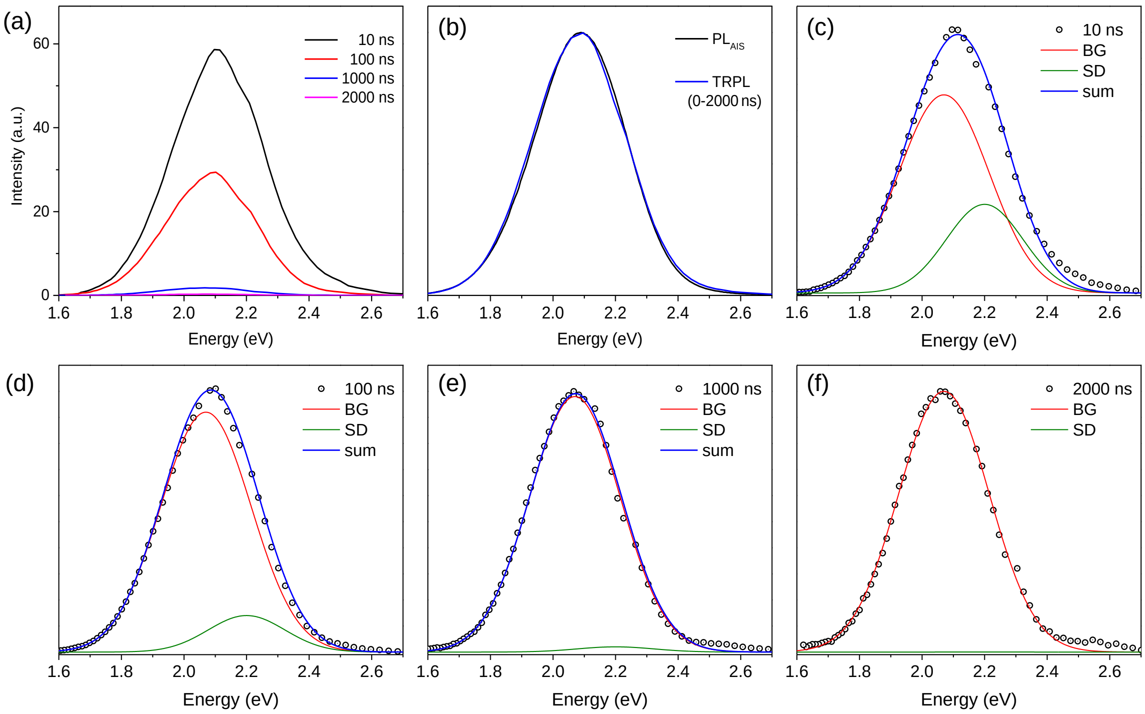

Fig. S3 (a) The TRPL spectra of AIS show the intensity decrease and red-shift of emission with increase in detection time. (b) The steady-state PL spectrum is almost identical to the integrated TRPL spectrum for the temporal range of 0-2,000 ns, which is normalized for better comparison. The TRPL spectra at the detection time of (c) 10 ns, (d) 100 ns, (e) 1,000 ns, and (f) 2,000 ns are decomposed into two bands (BG and SD) with the identical peak energies of the steady-state PL spectrum.

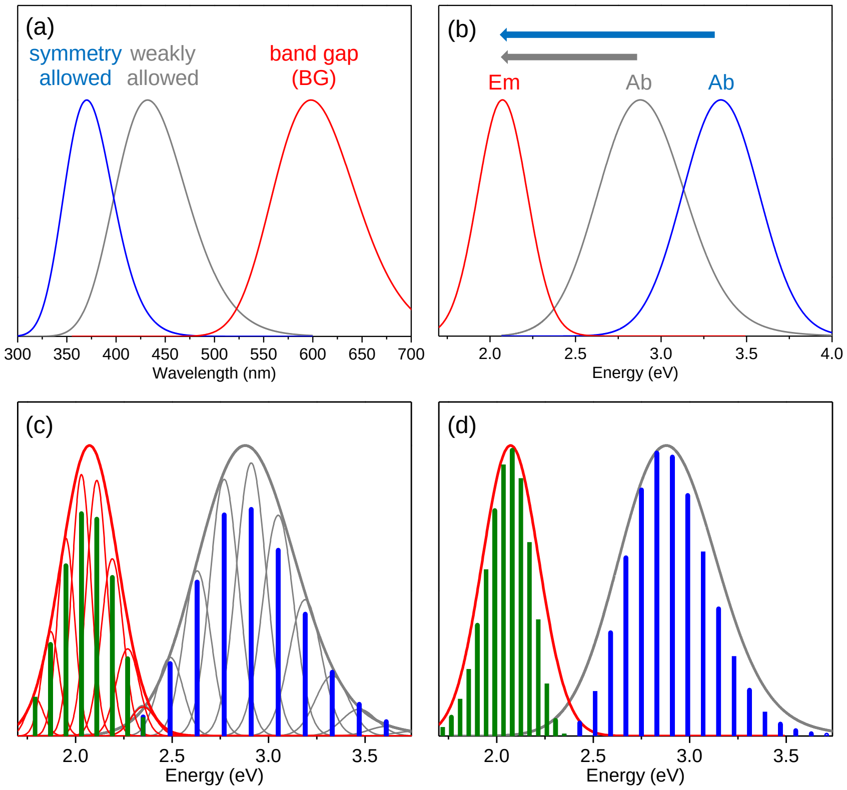

Fig. S4 The band gap emission (BG) is compared to the high-energy absorption (symmetry-allowed transition) and low-energy absorption (weakly-allowed transition) in (a) wavelength and (b) energy scale. The intensities are normalized for comparison. (c) The low-energy absorption (gray) and band gap emission (red) of AIS are decomposed into combination phonon modes, which are simulated with S = 4.1. The vertical bars indicate the relative intensities of phonon modes. (d) The spectral profiles of the low-energy absorption and band gap emission are simulated using single-phonon modes with S = 6.7.

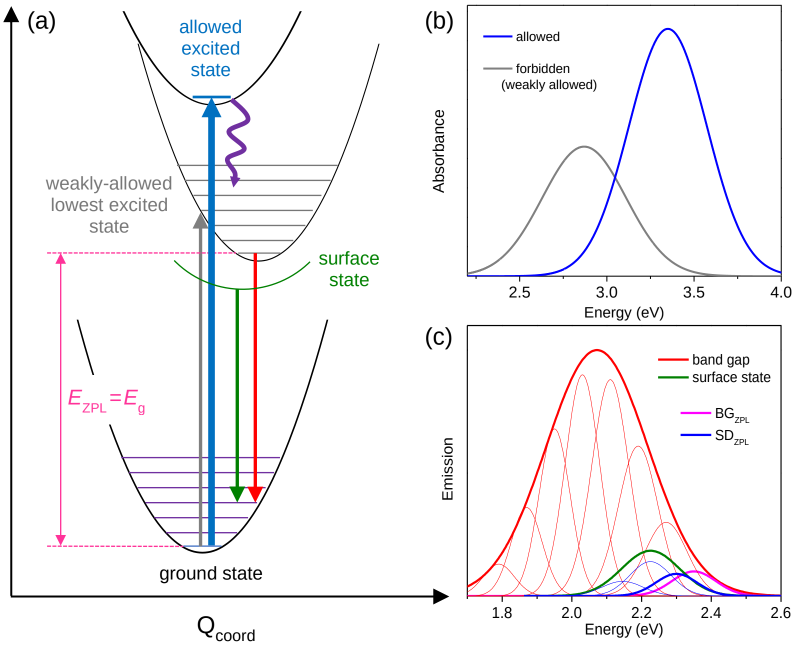

Fig. S5 (a) Schematic optical transitions along the coupled coordinate in AIS. The low-energy absorption (gray arrow) is the weakly-allowed transition, while the high-energy absorption (blue arrow) is the symmetry-allowed transition. The photoluminescence (red arrow) mainly takes place from the lowest-excited state to ground state, despite the major absorption to a higher-lying excited state, due to the fast relaxation (violet arrow). The energy of the zero-phonon line (E_{ZPL}) implies the band gap energy (E_g). The minor emission (green arrow) takes place from the surface states. (b) The energy and intensity of the low-energy absorption are compared to those of the high-energy absorption. (c) The E_{ZPL} of surface states is compared to that of band gap states.

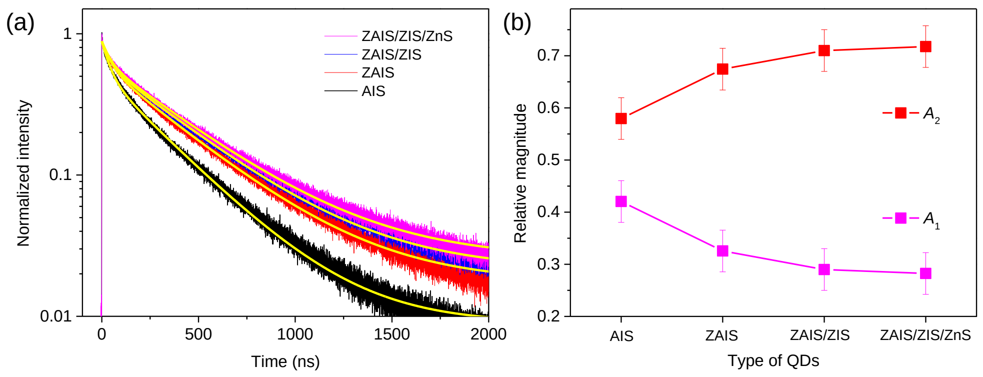

Fig. S6 (a) The normalized time-resolved PL profiles are fitted by the bi-exponential model (yellow lines). (b) The relative magnitudes of the fast component (A_1) and slow component (A_2) are obtained using fitting processes.

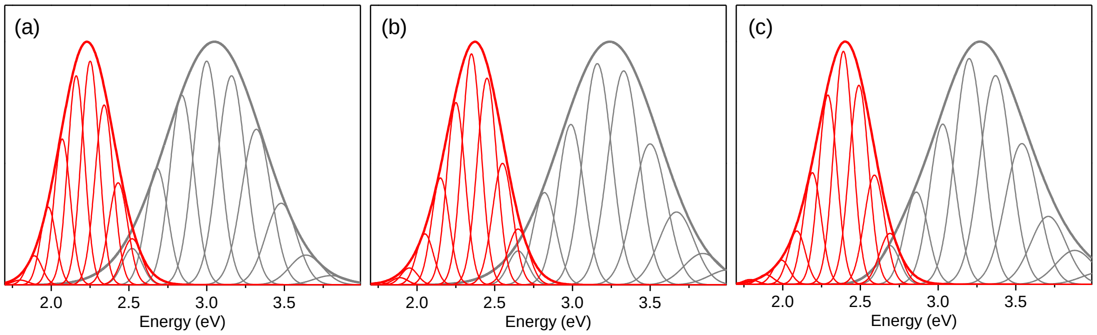

Fig. S7 The low-energy absorption (gray) and band gap emission (red) in (a) ZAIS, (b) ZAIS/ZIS, and (c) ZAIS/ZIS/ZnS are decomposed into progression of phonon modes.

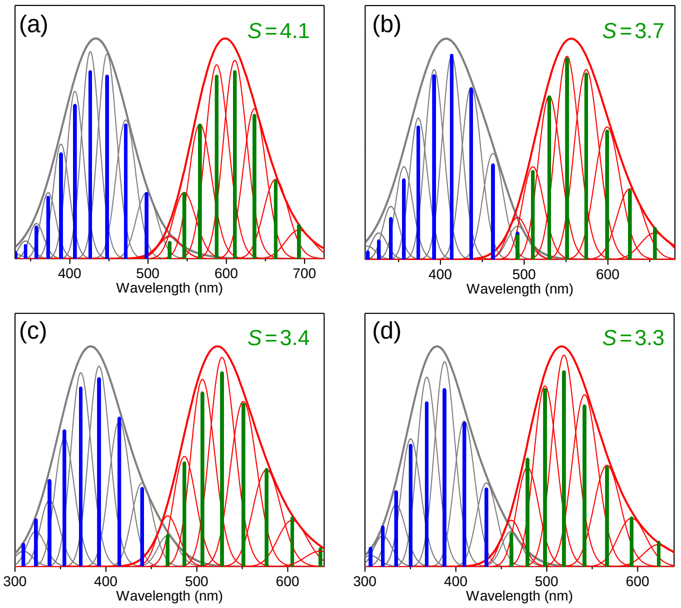

Fig. S8 The low-energy absorption (gray) and band gap emission (red) in (a) AIS, (b) ZAIS, (c) ZAIS/ZIS, and (d) ZAIS/ZIS/ZnS are decomposed into phonon modes. The decomposed bands are simulated with the combination mode using the configuration coordinate model. The Huang−Rhys factors of optical transitions (*S*) are also presented.

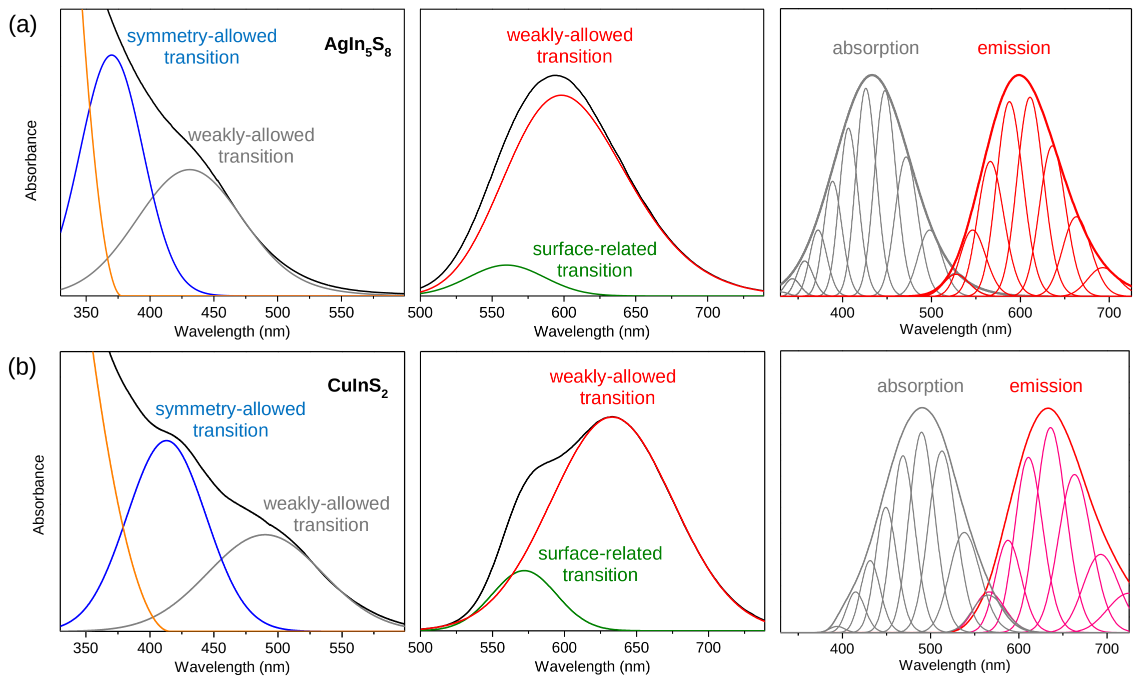

Fig. S9 The decomposed absorption and PL spectra of (a) AIS and (b) $CuInS_2$ QDs. The low-energy absorption (gray) and band gap emission (red) are decomposed into phonon modes.
